# HDAC1 and HDAC2 Are Involved in Influenza A Virus-Induced Nuclear Translocation of Ectopically Expressed STAT3-GFP

**DOI:** 10.3390/v17010033

**Published:** 2024-12-29

**Authors:** Jessica Leong, Matloob Husain

**Affiliations:** Department of Microbiology and Immunology, University of Otago, P.O. Box 56, Dunedin 9054, New Zealand

**Keywords:** Influenza A virus, innate antiviral response, acetylation, histone deacetylase, HDAC1, HDAC2, STAT3, GFP, nuclear translocation, confocal microscopy

## Abstract

Influenza A virus (IAV) remains a pandemic threat. Particularly, the evolution and increased interspecies and intercontinental transmission of avian IAV H5N1 subtype highlight the importance of continuously studying the IAV and identifying the determinants of its pathogenesis. Host innate antiviral response is the first line of defense against IAV infection, and the transcription factor, the signal transducer and activator of transcription 3 (STAT3), has emerged as a critical component of this response. Also, histone deacetylase 1 (HDAC1) and HDAC2 have been identified as important components of IAV-induced host innate antiviral response. Upon IAV infection, STAT3 is activated and translocated to the nucleus to initiate the transcription of innate response genes. Also, the HDAC1 and HDAC2 are localized to the nucleus. In this study, we sought to investigate the role of HDAC1 and HDAC2 in IAV-induced STAT3 nuclear translocation. We employed a quantitative confocal microscopy approach and analyzed the nuclear translocation of plasmid-expressed STAT3-GFP in IAV-infected cells depleted with the expression of HDAC1 or HDAC2. We found that the depletion of both HDAC1 and HDAC2 expression inhibits the IAV-induced nuclear translocation of STAT3-GFP. These findings will help elucidate the significance of the emerging role of acetylation in IAV infection and disease severity.

## 1. Introduction

Influenza A virus (IAV) is the prototypic member of the *Orthomyxoviridae* family and causes an acute febrile respiratory disease called influenza, commonly known as the ‘flu’ in humans. IAV has a broad host range and contains an error-prone negative-sense, single-stranded RNA genome in a segmented configuration. These characteristics enable constant circulation of IAV in nature and evolution of new IAV variants. Consequently, IAV continues to cause recurring seasonal epidemics and zoonotic outbreaks in the human population and remains a pandemic threat. Importantly, the avian IAV subtype H5N1 has become a major pandemic concern as it continues to evolve and infect humans and various animals and has increased its repertoire of hosts to cattle [1,2,3,4,5,6]. Therefore, it is critical to identify the determinants of IAV pathogenesis and design targeted anti-IAV strategies.

Host innate antiviral response is the first line of defense against IAV infection. Innate antiviral response is a complex network comprising a multitude of sensing and signaling molecules and pathways, which sense the IAV infection and initiate a cascade of signaling mechanisms to inhibit the infection [7]. The signal transducer and activator of transcription (STAT) family transcription factors play a pivotal role in host innate antiviral response. STATs are an integral component of the canonical Janus kinase (JAK)-STAT pathway, which, upon virus infection, is activated by cytokines like interferons (IFNs) and culminates in the expression of hundreds of interferon-stimulated genes (ISGs), which create an ‘antiviral state’ in host cells [7,8]. STATs are localized to the cytoplasm of uninfected cells in an inactivated form. However, upon virus infection, STATs are activated via phosphorylation by JAKs and bind interferon regulatory factor 9 (IRF9) to form the IFN-stimulated gene factor 3 (ISGF3) complex. The ISFG3 is then translocated to the nucleus and binds IFN-stimulated response elements (ISREs) of ISGs on the host genome and initiates the transcription of ISGs [7].

The STAT3 is one of the seven STATs known [9] and, depending on the virus, exhibits both proviral and antiviral properties [10]. During the IAV infection, STAT3 is activated and exhibits anti-IAV properties [11,12,13], though its role in IAV infection has not been greatly investigated. Notably, STAT3 is acetylated and interacts with class I histone deacetylase (HDAC), HDAC1 and HDAC1 and its “twin” HDAC2 regulate the STAT3 transcriptional activity under heterologous conditions [14,15,16]. Earlier, we demonstrated that both HDAC1 and HDAC2 inhibit IAV infection by promoting the innate antiviral response [17,18]. Furthermore, HDAC2 promotes STAT1 phosphorylation during the early stage of IAV infection [17], hence potentially its nuclear translocation in infected cells. Therefore, to further understand the antiviral mechanisms of HDAC1 and HDAC2, we sought to determine if HDAC1 and/or HDAC2 are involved in the IAV-induced nuclear translocation of STAT3. Herein, we demonstrate that, indeed, both HDAC1 and HDAC2 are involved in the nuclear translocation of STAT3 in IAV-infected cells.

## 2. Materials and Methods

### 2.1. Cells, Virus, and Infection

HeLa cells (ATCC CCL-2) were grown in Dulbecco’s Modified Eagle Medium (DMEM) (ThermoFisher Scientific, Waltham, MA, USA) supplemented with 10% fetal bovine serum (FBS, HyClone, Washington, DC, USA), penicillin-streptomycin (ThermoFisher), and L-Glutamine (ThermoFisher) at 37 °C under 5% CO_2_ atmosphere. Influenza virus A/Puerto Rico/8/1934(H1N1) was propagated in 10-day-old embryonated chicken eggs and titrated on MDCK cells.

For infection, the old culture medium was removed from the cells, and cells were washed twice with serum-free DMEM. Virus inoculum was made to the desired multiplicity of infection (MOI) by diluting the virus stock in serum-free DMEM. Cells were then incubated with virus inoculum at 35 °C for 1 h. The virus inoculum was then replaced with DMEM supplemented with 2% FBS and cells were returned to 35 °C for the desired incubation period.

### 2.2. Plasmid and Transfection

The plasmid pEGFP-N1-STAT3 [19] was a gift from Geert van den Bogaart (Addgene plasmid #111934) and plasmid pEGFP-N1 was gifted by Keith Ireton (University of Otago, New Zealand). Plasmids were propagated in *Escherichia coli* and plasmid DNA was extracted using a Plasmid Midi Kit (QIAGEN, Hilden, Germany) by following the manufacturer’s protocol.

For transfection, 1 microgram of plasmid DNA and 2 microliters of Lipofectamine 2000 (ThermoFisher) were diluted separately in OptiMEM I (ThermoFisher). After 5 min incubation at room temperature, the DNA and Lipofectamine 2000 solutions were mixed, and the mixture was incubated for 30 min at room temperature. Then, the old culture medium of cells was replaced with DNA and Lipofectamine 2000 mixture, and cells were incubated at 37 °C. After 4 h, the DNA and Lipofectamine 2000 mixture was replaced with DMEM supplemented with 10% FBS, and cells were returned to 37 °C for the desired incubation period.

### 2.3. RNA Interference

Predesigned small-interfering RNA (siRNA) targeting human HDAC1 gene (ID: SASI_Hs01_00079964) and HDAC2 gene (ID: SASI_Hs02_00332058) and a nontargeting MISSION Control siRNA were obtained from Sigma-Aldrich (St. Louis, MO, USA). For transfection, 5 nanomolars of siRNA and 2 microliters of Lipofectamine RNAiMAX (ThermoFisher) were diluted separately in OptiMEM I. After 5 min incubation at room temperature, the siRNA and Lipofectamine RNAiMAX solutions were mixed, and the mixture was incubated for 45 min at room temperature. During this incubation period, cells were split and diluted in DMEM to the desired number. Then, cells and the siRNA and Lipofectamine RNAiMAX mixture were combined and added to a cell culture plate. The cells were then incubated at 37 °C for the desired period.

### 2.4. Western Blotting

Cells were lysed in a buffer containing 50 mM Tris-HCl (pH 7.4), 150 mM NaCl, 0.5% sodium dodecyl sulfate (SDS), 0.5% sodium deoxycholate, 1% TritonX-100 and protease inhibitor cocktail (Roche, Basel, Switzerland), and total protein amount was quantified using a Pierce BCA protein assay kit (ThermoFisher). Samples containing an equal concentration of protein along with a pre-stained protein standard SeeBlue Plus 2 (ThermoFisher) were resolved on 8% or 10% Tris-Glycine SDS-polyacrylamide gel electrophoresis (PAGE). The protein was then transferred onto a nitrocellulose membrane (Cytiva, Washington, DC, USA), which was blocked with 5% non-fat milk in Tris-buffered saline (TBS). The membrane was probed with Mouse anti-STAT3 (BD Biosciences, Franklin Lake, NJ, USA), Mouse anti-NP (Clone 2F4, NR-19868 BEI Resources, NIAID, NIH), Mouse anti-HDAC1 (10E2), Mouse anti-HDAC2 (3F3) (Cell Signaling, Danvers, MA, USA), or Mouse anti-actin (Sigma-Aldrich, St. Louis, MO, USA) antibody followed by Goat anti-Mouse IgG conjugated with horseradish peroxidase (ThermoFisher). Protein bands were visualized by chemiluminescence using an ECL-Prime Western blotting substrate (Cytiva), and images were acquired on the Odyssey Fc imaging system (LI-COR, Lincoln, NE, USA).

### 2.5. Confocal Microscopy

Cells were fixed in 4% paraformaldehyde in phosphate-buffered saline (PBS) for 20 min at room temperature. Cells were then permeabilized with 0.2% Triton-X in PBS for 5 min at room temperature. Cells were stained with Mouse anti-HDAC1 (10E2) or anti-HDAC2 (3F3) antibody (Cell Signaling, Danvers, MA, USA) followed by Donkey anti-Mouse IgG conjugated with Alexa Fluor 594 (ThermoFisher) for 1 h and 30 min, respectively, at room temperature. Then, cells were stained with Goat anti-IAV nucleoprotein (NP) antibody (G150, a gift from Dr Richard Webby, St Jude Children’s Hospital, Memphis, TN, USA) followed by Donkey anti-Goat IgG conjugated with Alexa Fluor 647 (ThermoFisher) as above. All antibody dilutions were made in 10% FBS-PBS, and cells were washed at least 3 times between each antibody incubation. Subsequently, cells were stained with 1 mg/mL DAPI (4′,6-diamidino-2-phenylindole) in PBS for 10 min at room temperature. Finally, cells were visualized and imaged using a Zeiss LSM 900 Airyscan 2 confocal microscope.

### 2.6. Confocal Image Analysis

Confocal images were compiled and analyzed using ImageJ ver.1.54g and Arivis Vision 4D ver.4.1.2 software (Zeiss, Jena, Germany). Arivis is designed for advanced visualization, analysis, and management of biomedical image data. It consists of multiple programs or modules that work together to provide a comprehensive solution for image processing and analysis. Arivis is integrated with a deep learning-based algorithm called Cellpose for cell and nucleus segmentation [20]. Cellpose can precisely segment various cells from various image types, allowing for the acquisition of cell size and fluorescence intensities. Arivis was used to determine the mean pixel intensity of STAT3-GFP in the nucleus and cytoplasm and calculate the nuclear/cytoplasmic ratio of STAT3-GFP after various treatments.

### 2.7. Statistical Analysis

Statistical analyses were performed using Prism 10.3.1 (GraphPad, La Jolla, CA, USA). The *p*-values were calculated using two-way Analysis of Variance (ANOVA) employing Šídák’s multiple comparisons test. A *p*-value ≤0.05 was considered significant.

## 3. Results

To investigate the role of host HDAC1 and HDAC2 in IAV-induced nuclear translocation of transcription factor, STAT3, we employed the fluorescence microscopy approach. This has been the frequently used approach to study the nuclear-cytoplasmic trafficking of STAT3 and other STATs under various conditions, including viral infection [21,22,23,24,25,26,27,28,29,30,31,32,33,34]. For this, our strategy was to overexpress the STAT3-GFP fusion protein from a plasmid and then visualize the IAV-induced nuclear translocation of STAT3-GFP by confocal microscopy in the presence and absence of HDAC1 or HDAC2 expression. The nuclear translocation of plasmid-expressed STAT3 as well as other STATs, tagged with or without GFP, has been successfully demonstrated using different stimulants [21,22,23,24,25,26,27,28,29,30]. Moreover, immunolabelling of many endogenous proteins, including STAT3, can be challenging and depends on fixation and labeling procedures [35,36]. Therefore, we obtained a plasmid containing the STAT3-GFP fusion construct [19], transfected it to HeLa cells, and subsequently infected them with the influenza virus A/Puerto Rico/8/1934(H1N1), hereafter referred to as PR8. We used HeLa cells to obtain a better transfection efficiency, which we were unable to obtain with the physiologically relevant A549 cells in our hands. Although HeLa cells are not a physiologically relevant model for the influenza virus, they are amenable to infection with PR8 (and other IAV strains/subtypes) and have been used as a model to study influenza virus biology, particularly the intracellular trafficking [37,38,39,40,41,42,43,44]. First, we analyzed the overexpression of STAT3-GFP fusion protein by Western blotting using STAT3 antibody. We detected a prominent band (~120 kDa) representing the STAT3-GFP fusion protein (92 kDa STAT3 + 27 kDa GFP = 119 kDa) in both uninfected and infected cells transfected with the plasmid containing STAT3-GFP fusion construct (Figure S1). These data indicated that the plasmid is expressing the correct protein.

### 3.1. Plasmid-Expressed STAT3-GFP Translocates to the Nucleus in Response to Interferon Treatment

**Figure 1:**
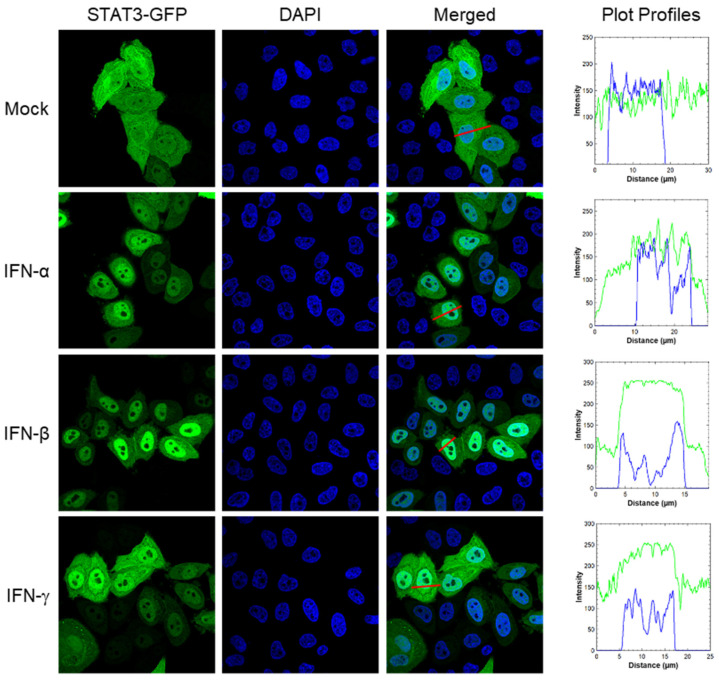
Plasmid-expressed STAT3-GFP translocates to the nucleus in response to interferon treatment. HeLa cells were transfected with plasmid pEGFP-N1-STAT3 for 48 h, and then treated with IFN-α, IFN-β, or IFN-γ (500 IU). After 1 h, cells were fixed and stained with DAPI (4′,6-diamidino-2-phenylindole), and then imaged using a confocal microscope under 40× magnification. Plot profiles of cells treated with IFN-α, IFN-β, and IFN-γ (next to respective rows) were generated using ImageJ 1.54g; red lines in Merged column indicate the profiled area; x-axes and y-axes on graphs represent distance and pixel intensity, respectively, along the lines; green and blue lines on graphs represent plot profiles of STAT3-GFP and DAPI, respectively.

Next, we analyzed whether plasmid-expressed STAT3-GFP is kinetically active and can translocate to the nucleus. For this, we transfected the cells with STAT3-GFP plasmid and treated them with IFN-α, IFN-β, or IFN-γ (obtained through BEI Resources, NIAID, NIH), and then stained them with DAPI to visualize the nuclei. Subsequently, cells were analyzed by confocal microscopy to visualize the intracellular distribution of STAT3-GFP. In mock-treated cells, STAT3-GFP was evenly distributed in the cytoplasm and the nucleus (though in some cells, it was more in the cytoplasm) (Figure 1, row 1). However, in the cells treated with IFNα (Figure 1, row 2), IFNβ (Figure 1, row 3), and IFNγ (Figure 1, row 4), STAT3-GFP was predominantly localized to the nucleus. Consistent with this distribution, the plot profile, which indicates the intensity of pixels (y-axis) along a line (x-axis) within an image, of STAT3-GFP in a mock-treated cell showed a flatter line across the x-axis that aligned partially with the plot profile of predominantly nuclear DAPI stain (Figure 1, row 1, column 4). In contrast, the plot profile of STAT3-GFP in cells treated with IFNα, IFNβ, or IFNγ showed a sharp peak in the middle of x-axes that aligned with the plot profile of DAPI (Figure 1, row 2–4, column 4). These data indicated that the plasmid-expressed STAT3-GFP is kinetically active, responds to IFNs, and follows the JAK-STAT pathway to the nucleus.

### 3.2. Plasmid-Expressed STAT3-GFP Translocates to the Nucleus in Response to IAV Infection

**Figure 2:**
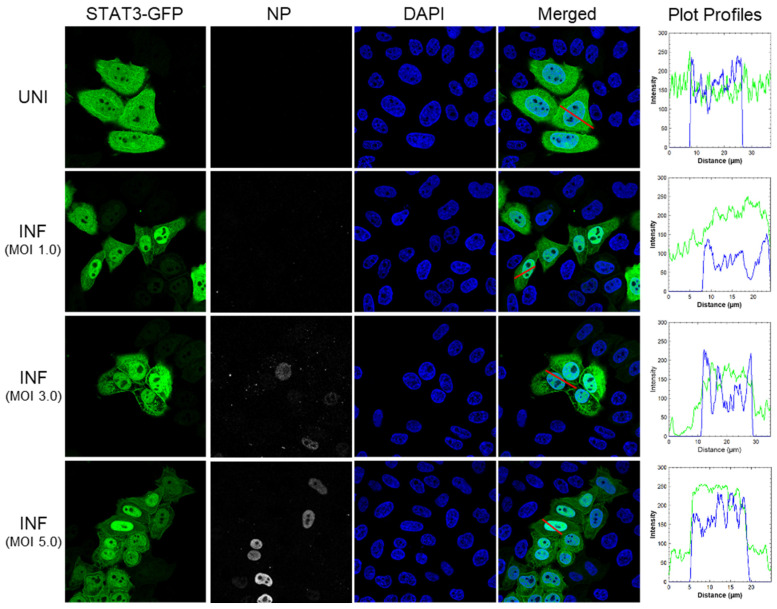
Plasmid-expressed STAT3-GFP translocates to the nucleus in response to IAV infection. HeLa cells were transfected with plasmid pEGFP-N1-STAT3 for 48 h, and then infected with influenza virus A/Puerto Rico/8/1934(H1N1), hereafter referred to as PR8, at the multiplicity of infection (MOI) of 1.0, 3.0, or 5.0. After 6 h, cells were fixed, permeabilized, and stained with Goat anti-IAV nucleoprotein (NP) antibody followed by Donkey anti-Goat IgG conjugated with Alexa Fluor 647. Subsequently, cells were stained with DAPI and imaged using a confocal microscope under 40× magnification. Plot profiles of uninfected (UNI) and infected (INF) cells (next to respective rows) were generated using ImageJ 1.54g; red lines in Merged column indicate the profiled area; x-axes and y-axes on graphs represent distance and pixel intensity, respectively, along the lines; green and blue lines on graphs represent plot profiles of STAT3-GFP and DAPI, respectively.

Next, we examined whether plasmid-expressed STAT3-GFP can translocate to the nucleus in response to IAV infection. For this, the STAT3-GFP plasmid-transfected cells were infected with PR8 at the multiplicity of infection (MOI) of 1.0, 3.0, or 5.0. After 6 h, cells were stained for nuclei and IAV nucleoprotein (NP) as an infection marker, and the nuclear translocation of STAT3-GFP was visualized by confocal microscopy. Like mock-treated cells in Figure 1, STAT3-GFP was evenly distributed in the cytoplasm and the nucleus of uninfected cells (Figure 2, row 1). Further, consistent with IFN treatment in Figure 1, STAT3-GFP was predominantly localized to the nucleus of infected cells regardless of the MOI (Figure 2, rows 2–4). Also, the STAT3-GFP plot profile, which was flatter across the x-axis and aligned partially with the DAPI plot profile in a mock-treated cell (Figure 2, row 1, column 5), exhibited sharp peaks in the middle of x-axes that aligned with the DAPI plot profiles in cells infected with all three MOIs (Figure 2, rows 2–4, column 5). However, the NP, which localizes to the nucleus at an early time of infection (6 h), could not be visualized in cells infected with the MOI of 1.0, potentially due to its low abundance at lower MOI. Nevertheless, together with IFN data, these data ascertained that HeLa cells expressing STAT3-GFP from a plasmid are a functional model to study the role of host HDAC1 and HDAC2 in the IAV-induced JAK-STAT pathway. Based on the results here, the MOI of 3.0 was selected for subsequent experiments.

### 3.3. The IAV-Induced Nuclear Translocation of STAT3-GFP Is Inhibited in HDAC1-Depleted Cells

**Figure 3:**
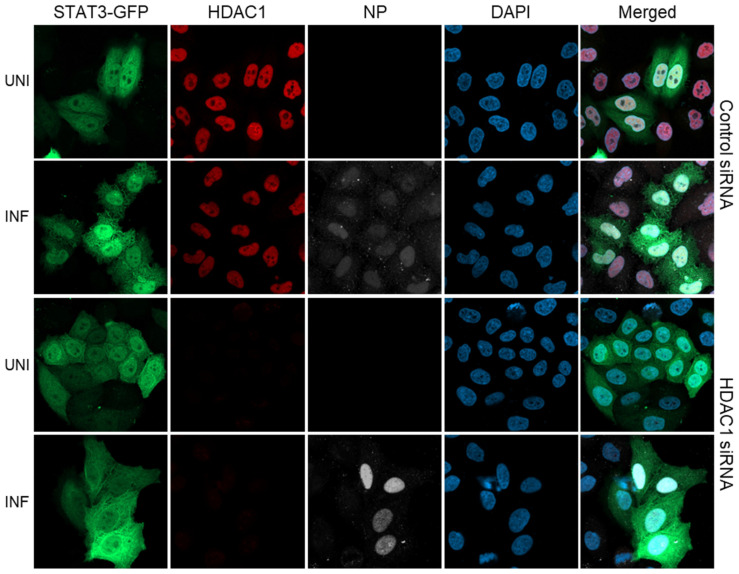
The IAV-induced nuclear translocation of STAT3-GFP is inhibited in HDAC1-depleted cells. HeLa cells were first transfected with either control siRNA or HDAC1 siRNA for 30 h and then transfected with plasmid pEGFP-N1-STAT3 for a further 30 h. Cells were then infected with PR8 at MOI of 3.0. After 6 h, cells were fixed, permeabilized, and stained with Mouse anti-HDAC1 antibody followed by Donkey anti-Mouse IgG conjugated with Alexa Fluor 594. Subsequently, cells were stained with Goat anti-NP antibody followed by Donkey anti-Goat IgG conjugated with Alexa Fluor 647. Finally, cells were stained with DAPI and imaged using a confocal microscope under 40× magnification. UNI, uninfected; INF, infected.

**Figure 4:**
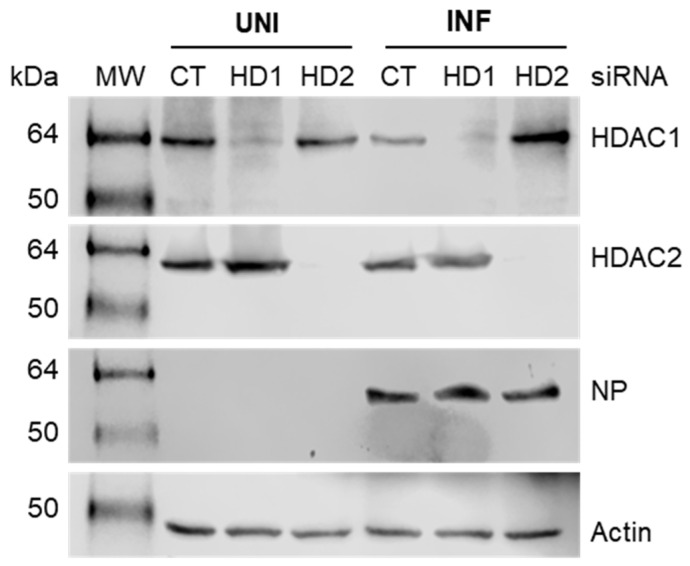
Western blots showing the siRNA-mediated depletion of HDAC1 and HDAC2 expression in uninfected and infected cells. HeLa cells were transfected with either control (CT), HDAC1 (HD1), or HDAC2 (HD2) siRNA for 30 h. Cells were then infected with PR8 at MOI of 3.0. After 6 h, HDAC1 (62 kDa), HDAC2 (60 kDa), NP (56 kDa), and Actin (42 kDa) were detected in total cell lysates by Western blotting. UNI, uninfected; INF, infected; kDa, kilodalton.

Using the above assay, we next investigated the role of HDAC1 in IAV-induced nuclear translocation of STAT3-GFP. For this, we first transfected HeLa cells with HDAC1 siRNA to deplete the expression of HDAC1, then transfected them with STAT3-GFP plasmid, and finally infected them with PR8. Subsequently, in addition to NP and nuclei, cells were stained for HDAC1 and visualized by confocal microscopy. Consistent with the above results, STAT3-GFP was equally distributed in the cytoplasm and nucleus of uninfected cells transfected with either Control siRNA (Figure 3, row 1) or HDAC1 siRNA (Figure 3, row 3). Further, STAT3-GFP was predominantly localized to the nucleus of infected cells transfected with the Control siRNA (Figure 3, row 2). However, in infected cells transfected with the HDAC1 siRNA, STAT3-GFP was equally distributed in the cytoplasm and nucleus (Figure 3, row 4). This indicated that the depletion of HDAC1 expression inhibited the IAV-induced translocation of STAT3-GFP from the cytoplasm to the nucleus. The lack of HDAC1 staining in the nucleus of HDAC1 siRNA-transfected uninfected cells as well as infected cells confirmed the depletion of HDAC1 expression (Figure 3, column 2). Normally, HDAC1 localizes to the nucleus, as visible here in Control siRNA-transfected uninfected cells as well as infected cells (Figure 3, column 2), and demonstrated elsewhere [45,46]. Nevertheless, we confirmed the depletion of HDAC1 expression in both uninfected and infected cells by Western blotting too (Figure 4, panel 1, lanes 3 and 6). Also, we confirmed the expression of HDAC2 in HDAC1-depleted cells to ensure that the depletion of HDAC1 does not interfere with the expression of HDAC2 (Figure 4, panel 2, lanes 3 and 6).

**Figure 5:**
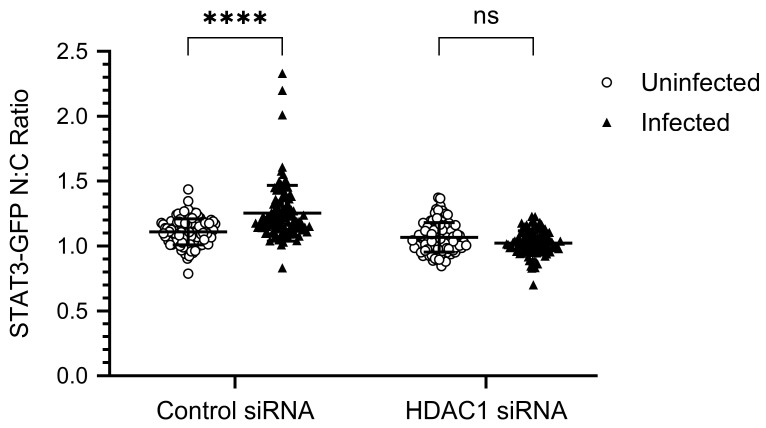
The nuclear/cytoplasmic ratio of STAT3-GFP is similar in HDAC1-depleted uninfected cells and HDAC1-depleted infected cells. The pixel density of STAT3-GFP in the nucleus and the cytoplasm of Control siRNA-transfected and HDAC1 siRNA-transfected uninfected as well as infected cells was quantified using Arivis Vision 4D ver.4.1.2 software (Zeiss) integrated with Cellpose. Then, STAT3-GFP pixel density per µm^2^ of nucleus was divided by STAT3-GFP pixel density per µm^2^ of cytoplasm to calculate the STAT3-GFP nuclear/cytoplasmic (N:C) ratio. The data presented are the Mean ± SD of N:C ratio of cells imaged across three biological replicates; n = 102 (36 from each replicate). The *p*-value was calculated using two-way Analysis of Variance (ANOVA) employing Šídák’s multiple comparisons test. **** *p* = <0.0001, ns, not significant.

To quantify the change in IAV-induced nuclear localization of STAT3-GFP between Control siRNA-transfected and HDAC1 siRNA-transfected cells, we quantified the pixel density of STAT3-GFP in the nucleus and the cytoplasm of Control siRNA-transfected and HDAC1 siRNA-transfected uninfected as well as infected cells. For this, we used Arivis Vision 4D ver.4.1.2 software (Zeiss) integrated with Cellpose [20]. The Cellpose is a cell and nucleus segmentation algorithm that can precisely segment cells from various image types, allowing for the acquisition of cell size and pixel intensities. Then, STAT3-GFP pixel density per µm^2^ of the nucleus was divided by STAT3-GFP pixel density per µm^2^ of cytoplasm to calculate the STAT3-GFP nuclear/cytoplasmic (N:C) ratio. According to this method, consistent with confocal data, the N:C ratio of STAT3-GFP was significantly higher (*p* = <0.0001) in Control siRNA-transfected infected cells (1.253) than in Control siRNA-transfected uninfected cells (1.109) (Figure 5). However, there was no significant change in the N:C ratio of STAT3-GFP between HDAC1 siRNA-transfected uninfected cells (1.067) and HDAC1 siRNA-transfected infected cells (1.024) (Figure 5). These data affirmed that HDAC1 is positively involved in the translocation of STAT3-GFP from cytoplasm to the nucleus in IAV-infected cells.

Although GFP and its modified form, enhanced GFP (EGFP), used here, lack a nuclear localization signal, owing to their smaller size (27 kDa), they can translocate to the nucleus by passive diffusion through nuclear pores [47]. Therefore, we next ascertained that the EGFP in the STAT3-GFP fusion protein was not influencing the nuclear translocation of STAT3-GFP and skewing the results obtained here. For this, we basically repeated the confocal experiment and calculated the N:C ratio as above in Figure 3 and Figure 5, respectively, but by transfecting the plasmid expressing EGFP alone. We found that, in uninfected cells transfected with the Control siRNA, EGFP was localized less in the cytoplasm and more in the nucleus (Figure S2, row 1). However, such EGFP distribution did not change after the infection and/or the depletion of HDAC1 expression (Figure S2, rows 2–4). Similarly, there was no significant change in the N:C ratio of EGFP between Control siRNA-transfected uninfected cells (1.291) and infected cells (1.295) and HDAC1 siRNA-transfected uninfected cells (1.305) and infected cells (1.28) (Figure S3). These data indicate that EGFP in STAT3-GFP has no or negligible impact on the STAT3-GFP nuclear translocation, and STAT3-GFP data presented above represent the true effects of IAV infection and HDAC1 expression.

### 3.4. The IAV-Induced Nuclear Translocation of STAT3-GFP Is Inhibited in HDAC2-Depleted Cells

**Figure 6:**
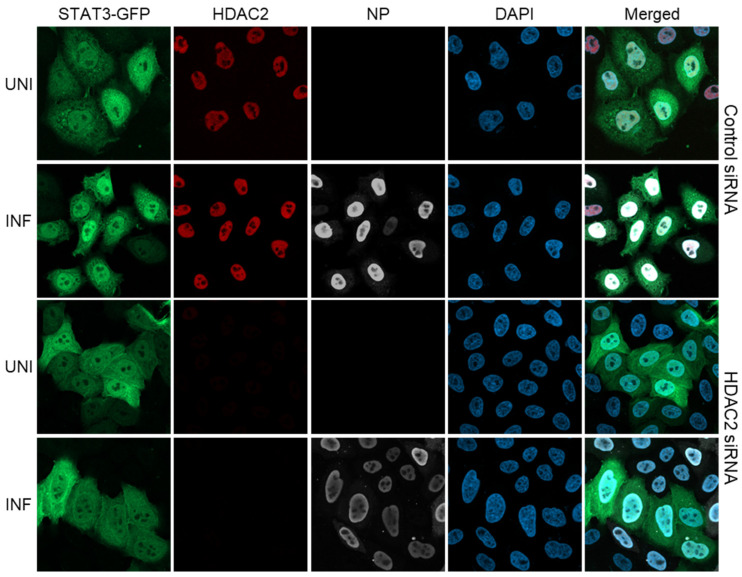
The IAV-induced nuclear translocation of STAT3-GFP is inhibited in HDAC2-depleted cells. HeLa cells were first transfected with either Control siRNA or HDAC2 siRNA for 30 h and then transfected with plasmid pEGFP-N1-STAT3 for a further 30 h. Cells were then infected with PR8 at MOI of 3.0. After 6 h, cells were fixed, permeabilized, and stained with Mouse anti-HDAC2 antibody followed by Donkey anti-Mouse IgG conjugated with Alexa Fluor 594. Subsequently, cells were stained with Goat anti-NP antibody followed by Donkey anti-Goat IgG conjugated with Alexa Fluor 647. Finally, cells were stained with DAPI and imaged using a confocal microscope under 40× magnification. UNI, uninfected; INF, infected.

Using identical conditions and parameters as HDAC1, we also investigated the role of HDAC2 in IAV-induced nuclear translocation of STAT3-GFP. For this, HeLa cells were transfected with HDAC2 siRNA and STAT3-GFP plasmid, subsequently infected with PR8, stained for HDAC2 and other markers, and then visualized by confocal microscopy as above. Like in Figure 3, STAT3-GFP was equally distributed in the cytoplasm and nucleus of uninfected cells transfected with either Control siRNA (Figure 6, row 1) or HDAC2 siRNA (Figure 6, row 3), but was largely localized to the nucleus of infected cells transfected with the Control siRNA (Figure 6, row 2). However, in infected cells transfected with HDAC2 siRNA, STAT3-GFP was equally distributed in the cytoplasm and nucleus (Figure 6, row 4). This indicated that, like HDAC1, the depletion of HDAC2 expression also inhibited the IAV-induced translocation of STAT3-GFP from the cytoplasm to the nucleus. As shown in Figure 6, column 2, and elsewhere [46], the HDAC2 also localizes to the nucleus, and lack of HDAC2 staining in HDAC2 siRNA-transfected uninfected as well as infected cells confirmed the depletion of HDAC2 expression (Figure 6, column 2). Furthermore, Western blotting confirmed the depletion of HDAC2 expression in both uninfected and infected cells too (Figure 4, panel 2, lanes 4 and 7). Also, the depletion of HDAC2 did not affect (albeit slightly increased) the expression of HDAC1 (Figure 4, panel 1, lanes 4 and 7).

**Figure 7:**
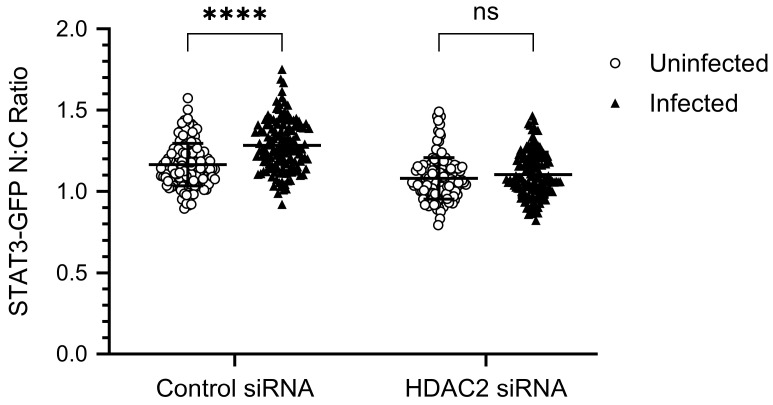
The nuclear/cytoplasmic ratio of STAT3-GFP is similar in HDAC2-depleted uninfected cells and HDAC2-depleted infected cells. The pixel density of STAT3-GFP in the nucleus and the cytoplasm of Control siRNA-transfected and HDAC2 siRNA-transfected uninfected as well as infected cells was quantified using Arivis Vision 4D ver.4.1.2 software (Zeiss) integrated with Cellpose. Then, STAT3-GFP pixel density per µm^2^ of nucleus was divided by STAT3-GFP pixel density per µm^2^ of cytoplasm to calculate the STAT3-GFP N:C ratio. The data presented are the Mean ± SD of N:C ratio of cells imaged across three biological replicates; n = 138 (46 from each replicate). The *p*-value was calculated using two-way ANOVA employing Šídák’s multiple comparisons test. **** *p* = <0.0001; ns, not significant.

The changes in IAV-induced nuclear localization of STAT3-GFP between Control siRNA-transfected and HDAC2 siRNA-transfected cells were quantified and the STAT3-GFP N:C ratio was calculated as above in Figure 5. As above, the N:C ratio of STAT3-GFP was significantly higher (*p* = <0.0001) in Control siRNA-transfected infected cells (1.283) than in Control siRNA-transfected uninfected cells (1.165) (Figure 7). However, there was no significant change in the N:C ratio of STAT3-GFP between HDAC2 siRNA-transfected uninfected cells (1.080) and HDAC2 siRNA-transfected infected cells (1.103) (Figure 7). Together with the above data, these data confirmed that both HDAC1 and HDAC2 positively regulate the translocation of STAT3-GFP from cytoplasm to the nucleus in IAV-infected cells.

## 4. Discussion

By using a quantitative confocal microscopy approach, we have demonstrated herein that anti-IAV host factors HDAC1 and HDAC2 are involved in the translocation of STAT3 to the nucleus in IAV-infected cells. In addition to STAT1 and STAT2 [48,49], STAT3 has emerged as an important transcription factor for the induction of host innate antiviral response to IAV infection [12]. STAT3 is expressed at a high level in IAV patients [13]. Furthermore, STAT3 is phosphorylated and activated in response to the IAV infection and inhibits IAV replication [11,12]. In addition, STAT3 is translocated to the nucleus in response to IFN-α treatment [16]. Consistent with this, we found herein that the plasmid-expressed STAT3 was signally and kinetically active and translocated to the nucleus in response to IFN-α, IFN-β, or IFN-γ treatment as well as IAV infection.

To demonstrate the IAV-induced STAT3 nuclear translocation and the role of HDAC1 and HDAC2 in such translocation, we used an early infection timepoint, 6 h. This is because STAT3 is activated within a couple of hours of IAV infection and its activation peaks within 6 h of IAV infection [12]. As infection progresses, the level of activated STAT3 is gradually decreased, and its reduced amounts are detected in IAV-infected cells at later infection timepoints due to IAV antagonizing the STAT3 activity [50,51,52]. Likewise, IAV also antagonizes HDAC1 and HDAC2, and their levels are decreased in infected cells at the later infection timepoints [17,18]. Therefore, the visualization of STAT3 nuclear translocation in the presence or absence of HDAC1 or HDAC2 expression at the 6 h infection timepoint represents its accurate activation window. Such visualization at later infection timepoints could have skewed the results and the effect of HDAC1 and HDAC2 on IAV-induced STAT3 nuclear translocation.

The inhibition of IAV-induced STAT3 translocation to the nucleus in the absence of HDAC1 or HDAC2 expression is consistent with our earlier findings and working hypothesis. Earlier, we demonstrated that an HDAC inhibitor (trichostatin A) reduces the IAV-induced phosphorylation of STAT1 and subsequent expression of ISGs [18]. Similarly, the depletion of HDAC2 expression reduces the IAV-induced phosphorylation of STAT1 and expression of an ISG [17]. Also, the data presented here are consistent with the findings by others that showed the requirement of deacetylase activity for induction of antiviral response [53]. Based on our and others’ findings, our working hypothesis is that acetylation is pro-IAV, and the HDACs are anti-IAV factors, and the latter exert their antiviral function by promoting host innate anti-IAV response [54]. Therefore, the inhibition of IAV-induced STAT3 nuclear translocation in HDAC1- or HDAC2-depleted cells conforms to this hypothesis and indicates that HDAC1 and HDAC2 promote the activation of STAT3 in IAV-infected cells. However, it is yet to be determined whether, in IAV-infected cells, HDAC1 and/or HDAC2 regulate the STAT3 activation directly, e.g., by deacetylating STAT3, or indirectly, e.g., by deacetylating other cytoplasmic or nuclear proteins associated with STAT3. An almost identical result obtained here vis-à-vis the STAT3 nuclear translocation in HDAC1- and HDAC2-depleted cells is not unexpected. This is because HDAC1 and HDAC2 are believed to have evolved from a single gene via gene duplication, share ~82% amino acid homology, and exhibit functional redundancy with some independent functions [55]. Furthermore, both HDAC1 and HDAC2 are critical components of multiple complexes [56]. If one of these complexes is involved in the nuclear translocation of STAT3, then the depletion of either HDAC1 or HDAC2 will interfere with the integrity and function of that complex to regulate STAT3 nuclear translocation.

Nevertheless, it is intriguing how the nucleus-localized proteins HDAC1 and HDAC2 promote the nuclear translocation of STAT3, which is activated in the cytoplasm. One possibility is that HDAC1 and HDAC2 promote the capture or retention of activated STAT3 in the nucleus after its transport from the cytoplasm. Evidently, both HDAC1 and HDAC2 are an integral component of several multiprotein complexes, namely Sin3A, NuRD, and CoREST, which reside in the nucleus [56], and the Sin3A complex is involved in the host innate response against IAV infection [57]. Future investigations should focus on this aspect of HDAC1/2 and STAT3 interplay in IAV-infected cells. Also, future studies should focus on investigating the role of HDAC1 and HDAC2 in the expression of STAT3-dependent ISGs like PKR, OAS2, Mx, and ISG15 in IAV-infected cells [11]. The findings from these studies will help elucidate the significance of acetylation in IAV infection and flu disease severity.

## Data Availability

The original contributions presented in the study are included in the article/supplementary material, further inquiries can be directed to the corresponding author.

## Supplementary Materials

The following supporting information can be downloaded at: https://www.mdpi.com/article/10.3390/v17010033/s1, Figure S1: Overexpression of STAT3-GFP fusion protein from plasmid; Figure S2: The intracellular distribution of EGFP remains unchanged in control siRNA-transfected and HDAC1 siRNA-transfected uninfected or infected cells; Figure S3: The nuclear/cytoplasmic ratio of EGFP is similar in control siRNA-transfected and HDAC1 siRNA-transfected uninfected or infected cells.
