# Supplementary material for: HDAC1 and HDAC2 Are Involved in Influenza A Virus-Induced Nuclear Translocation of Ectopically Expressed STAT3-GFP
Source: Viruses. 2024 Dec 29;17(1):33. doi: 10.3390/v17010033 (PMC11769489; doi:10.3390/v17010033)
Supplement: Supplementary file 1 [file viruses-17-00033-s001.zip › viruses-3286329-supplementary.pdf]

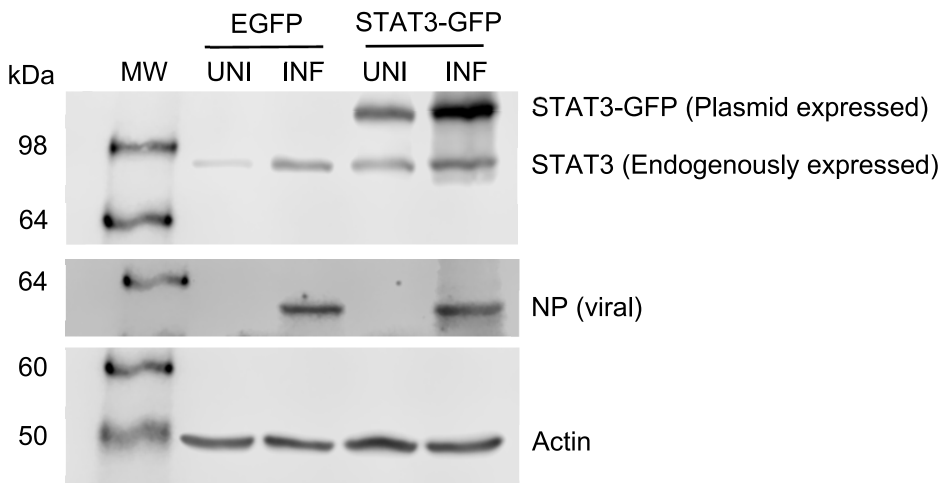

Figure S1. Overexpression of STAT3-GFP fusion protein from plasmid. HeLa cells were transfected with plasmid pEGFP-N1 or pEGFP-N1-STAT3 for 48 hours, then infected with influenza virus A/Puerto Rico/8/1934(H1N1), hereafter referred to as PR8, at an MOI of 3.0. After 6 h, STAT3-GFP (~120 kDa), STAT3 (92 kDa), NP (56 kDa) and Actin (42 kDa) were detected in total cell lysates by western blotting. UNI, uninfected; INF, infected; kDa, kilodalton.

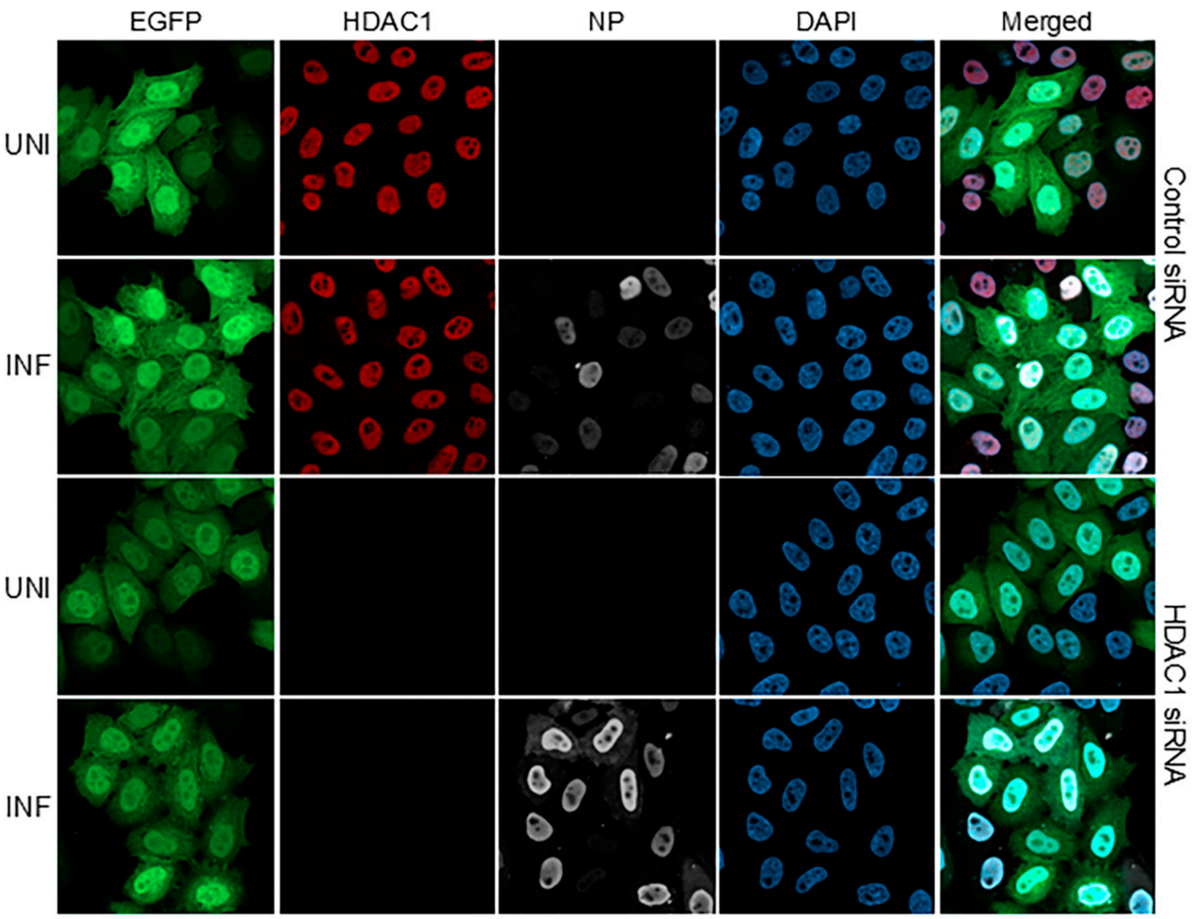

Figure S2. The intracellular distribution of EGFP remains unchanged in control siRNA-transfected and HDAC1 siRNA-transfected uninfected or infected cells. HeLa cells were first transfected with either Control siRNA or HDAC2 siRNA for 30 h and then transfected with plasmid pEGFP-N1 for further 30 h. Cells were then infected with PR8 at MOI of 3.0 for 6 h. Subsequently, cells were fixed, permeabilised, and stained with Mouse anti-HDAC1 antibody followed by Donkey anti-Mouse IgG conjugated with Alexa Fluor 594. Cells then were stained with Goat anti-NP antibody followed by Donkey anti-Goat IgG conjugated with Alexa Fluor 647. Finally, cells were stained with DAPI and imaged using confocal microscope under 40X magnification. UNI, uninfected; INF, infected.

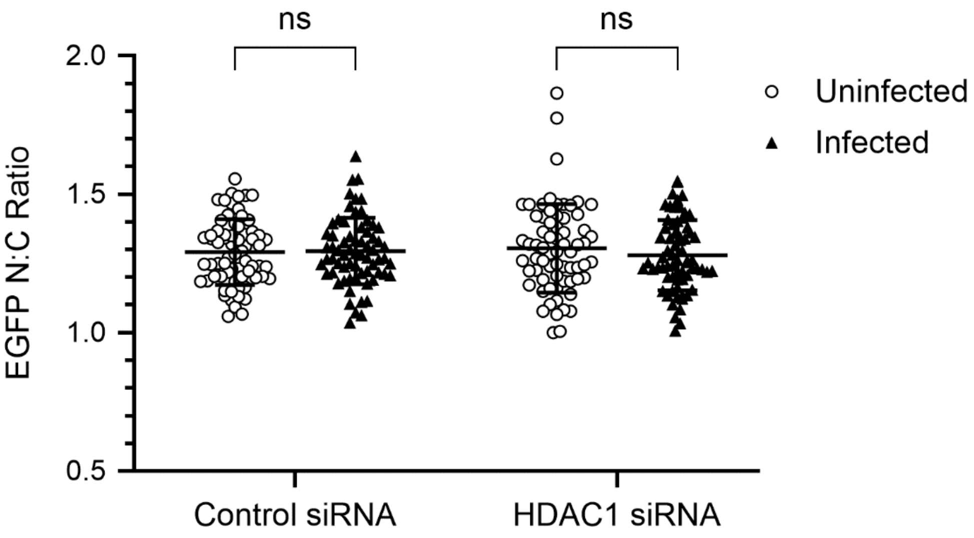

Figure S3. The nuclear-cytoplasmic ratio of EGFP is similar in control siRNA-transfected and HDAC1 siRNA-transfected uninfected or infected cells. The pixel density of EGFP in the nucleus and cytoplasm of Control siRNA-transfected and HDAC1 siRNA-transfected uninfected and infected cells was quantified using Arivis Vision 4D ver.4.1.2 software (Zeiss) integrated with Cellpose. Then, EGFP pixel density per μm^2 of nucleus was divided by EGFP pixel density per μm^2 of cytoplasm to calculate the EGFP nuclear-cytoplasmic (N:C) ratio. The data presented are the Mean ± SD of N:C ratio of cells imaged across one replicate (n = 67). The P value was calculated using two-way Analysis of Variance (ANOVA) employing Šídák's multiple comparisons test. ns, not significant.
